# Mothers’ dietary practices in the Amoron’i Mania region Madagascar

**DOI:** 10.11604/pamj.2018.30.76.15140

**Published:** 2018-05-29

**Authors:** Lantonirina Ravaoarisoa, Hasina Raherimandimby, Julio Rakotonirina, Jean de Dieu Marie Rakotomanga, Michèle Wilmet Dramaix, Philippe Donnen

**Affiliations:** 1 Institut National de Santé Publique et Communautaire, Antananarivo, Madagascar; 2 Faculté de Médecine d’Antananarivo, Madagascar; 3 Ecole de Santé Publique de l’Université Libre de Bruxelles, Belgique; 4 Centre Hospitalier Universitaire de Fianarantsoa, Madagascar

**Keywords:** Dietary pattern, mother, food frequency consumption, dietary diversity

## Abstract

**Introduction:**

Madagascar has one of the highest prevalence's of malnutrition worldwide. Dietary practice is an important element to consider in the fight against malnutrition. This study aims to describe mothers' dietary patterns and dietary diversity and to identify characteristics associated with this dietary diversity.

**Methods:**

A cross sectional study was carried-out among 670 non-pregnant mothers aged 18 to 45, who had delivered more than 6 months earlier and were living in the Amoron'i Mania region of Madagascar. The study was conducted during the post-harvest period. A food frequency questionnaire were used to assess the dietary pattern and the women's dietary diversity score was established from the 24-hour recall data.

**Results:**

Almost all (99%) of mothers ate rice every day and 59% ate green leaves. Fifty three percent of mothers had consumed fruit less than once per week, 55% for legumes, 67% for vegetables and 91% for meat. Dietary diversity score ranged from 1 to 7 and 88% of mothers had a low dietary diversity score (<5). On multivariate analysis, factors significantly associated with low dietary diversity were: low education level (AOR=3.80 [1.58-9.02], p=0.003), parity higher than 3 (AOR=2.09 [1.22-3.56], p=0.007), birth interval ≥ 24 months (AOR=4.01 [2.08-7.74], p<0.001), rice production availability ≤ 6 months (AOR=2.33 [1.30-4.17], p=0.013), low attendance at market (AOR=4.20 [1.63-10.83], p<0.001) and low movable property possession score (AOR=4.87 [2.15-11.04], p<0.001).

**Conclusion:**

Mother's experience poor diet diversity. Unfavorable socioeconomic conditions are associated with this poor food diversification.

## Introduction

Food consumption pattern is a key element to take into account in the fight against malnutrition as food is the main determinant of nutritional status. Various factors influence this practice such as biological, economic, social, physical, psychological, cultural and environmental factors [1-3]. In poor countries, the population's dietary practice mainly depends on food security, which refers to food availability stability and food access [4]. In countries where household food insecurity and undernutrition are purveying issues, it is necessary to know the population's dietary intake. Madagascar is one of the 20 countries with a high prevalence of undernutrition [5]. The prevalence of both maternal and child malnutrition remains high, and it has not been improving for several years. The results of the Demographic and Health Survey (DHS) conducted in 2008-2009 reported a prevalence of malnutrition (Body Mass Index, BMI <18.5 kg/m²) among 26.7% of women of reproductive age, that anemia was found in 35% of women and that 48% of children suffered from chronic malnutrition [6]. Food insecurity affects a high proportion of the population of Madagascar, especially in rural areas. In 2013, 58% of the population had a low quantity diet and 60% had a diet of extremely low quality [7]. In this context of food insecurity, there is a paucity of information about dietary practice, which is essential to understand in order to resolve the problem of undernutrition. So, it is necessary to broaden knowledge on dietary pattern and the factors that influence them. The aim of this study is to describe mothers' dietary pattern and dietary diversity and identify characteristics associated with dietary diversity.

## Methods

**Study site**: The study was conducted in the Amoron'i Mania region, one of Madagascar's 22 regions and located in the central highlands of the country. The Region comprises 4 health districts, 53 communes, of which 52 are located in rural areas, and about 580,000 inhabitants. Agriculture, mainly subsistence, was the main activity of the population [7]. This region has the highest undernutrition (BMI<18.5 kg/m²) prevalence among women of reproductive age in Madagascar. It was estimated at 41.6% in the last DHS results in 2008-2009 [6]. The study focused on rural areas where undernutrition issues are much more prevalent [6].

**Study population**: A cross sectional study was carried out. The study population included non-pregnant mothers between 18 and 45 years of age who had given birth more than 6 months earlier. Mothers under 18 years old were not included because of the difficulty in getting the guardian's consent. Mothers over 45 years old were not included in order to include mothers within the reproduction age group, knowing that the fertility rate is very low for women between 45 and 49 years old [6]. To ensure the validity of weight measurements, we also excluded women who had given birth within the last 6 months. Indeed, there is a gradual reduction of pregnancy weight gain and stabilization of mothers' weight around the sixth month after deliveries [8,9].

**Sampling**: A two-stage cluster sampling was used. The first stage aimed at selecting 30 “fokotany” (the smallest administrative structure) out of the 760 in the Region. It was done by systematic random sampling. The second stage was used to select, for each “fokotany”, eligible mothers from a list established by community workers. This was done by simple random sampling. Sample size was calculated on the basis of the national prevalence of maternal undernutrition (27%), with a 5% margin of error, 95% confidence level and a design effect of 2 [10]. The sample size was estimated to be 606. Twenty-one subjects per cluster therefore had to be included. During data collection, 670 women were actually interviewed.

**Data collection**: Data collection was conducted in July and August 2015, during the post-harvest (rice harvest) period in the region. Regarding mothers' dietary practice, two methods were used: a 24-hour recall method for mothers' dietary diversity assessment and a food frequency questionnaire for measuring dietary practice during the post-harvest period. For the 24-hour recall, interviewers asked and established the list of all food eaten by the mother the day before the survey. To minimize omissions, they focused on food intake based on the pre-established list to assess women's dietary diversity. Information about the place and the people with whom the mother ate the meal as well as the occurrence of an unusual event during the day before the survey was collected to detect unusual food consumption [11]. As for the food frequency questionnaire, it takes into account the last 3 months before the survey. This rather long period of time was chosen to have an idea about diets during the harvest period and to take rarely consumed food (less than once per month) into account. Nutritional status was assessed by use of the following anthropometric measurements: height, Body Mass Index (BMI), Mid Upper Arm Circumference (MUAC) and by hemoglobin measure for anemia. Interviewers were recruited locally. They had a bachelor's degree and were fluent in the local dialect. Samples management and transfers were dealt with by nurses working in the laboratory. Data collectors received training according to their mission. The investigator, a technician from the Nutrition Department of the Ministry of Public Health and the nutrition regional manager supervised the data collection. The study was approved by Malagasy Ministry of Health's Ethics Committee.

### Variables

***Dietary practice***: Dietary diversity score: for each woman, the consumption of 10 food groups was established from the 24-hour recall data. The consumption of one or more foods in one group was worth 1 point and the maximum score was 10. Afterwards, the score was categorized into two groups: lower than 5 and higher than or equal to 5. A score of five represents the lower limit which assures the qualitative nutrition need [12]. List of 10 food groups for the dietary diversity score of women [12]: 1) All starchy staples; 2) Beans and peas; 3) Nuts and seeds; 4) All dairy; 5) Flesh foods (including organ meat and miscellaneous small animal protein); 6) Eggs; 7) Vitamin A-rich dark green leafy vegetables; 8) Other vitamin A-rich vegetables and fruits; 9) Other vegetables; 10) Other fruits. Food consumption frequency was categorized into 4 groups: none, less than once a month, 1 to 4 times per month and more than once a week.

***Nutritional status***: The BMI was calculated by dividing weight in kilograms by height in square meters. Hemoglobin rate was adjusted for altitude [13]. World Health Organization (WHO) defined standards were used to identify undernourished women, i.e. BMI below 18.5 kg/m², height below 145cm, and MUAC below 220cm and women affected by anemia (hemoglobin < 120 g/l) [8,13].

***Social profile***: Mother's age, education level (last school year taken into account), occupation, marital status and husband's occupation were collected. Information about number of pregnancies, parity, number of children aged less than 5 years old, breastfeeding and birth interval were collected. The birth interval was calculated for the last two deliveries within the last five years. Afterwards, that interval was grouped using a 24-months threshold. Mothers who did not have two childbirths within the last five years and three primiparous women were classified in the 24 months or more group.

***Economic profile***: Three indicators of economic level were created considering the possession of household goods. We used the DHS Madagascar list to establish our list of goods. The first indicator refers to possession of movable property (furniture, radio, TV, telephone, bicycle, etc.), the second refers to possession of farming equipment and the third to possession of farm animals. The corresponding scores for these properties were established by principal components analysis (PCA). The scores were categorized into three groups (high, medium and low) based on values as close as possible to the tertiles. The period (number of months) in which a household consumes its annual rice production was also collected. It was divided in two groups with a 6 months threshold. Rice production is considered as an indicator of food security in study areas and can reflect the economic level of households. Two categorical variables on market access were defined: the number of times the household goes to the market (3 categories) and the time it takes to go to the nearest market (3 categories).

**Data analysis**: Stata/IC 13.1 (StataCorp LP, College Station, USA) software was used to analyze the data. In bivariate analysis using logistic regression, the link between low dietary diversity (Dietary Diversity Score <5) and other variables was estimated by the Odds Ratio (OR) with its confidence interval and chi-square or chi-square for trend tests were used. Variables with a p-value <0.20 in bivariate analysis were considered for inclusion in a logistic regression model. A stepwise backward method was used for selection of statistically significant covariates. Variables with more than two categories were transformed into indicators. The backward procedure used to select variables in the final model was based on the likelihood ratio. The adequacy of the final model was checked using Hosmer Lemeshow test. The adjusted OR (AOR) and their 95% confidence intervals were computed from the final logistic model. The significance level (p-value) was set at 0.05.

## Results

### Sample description

**Mothers' characteristics**: **Table 1** shows the description of 670 mothers included in the study. Fourteen percent of them were illiterate. Nearly 3 out of 4 of mothers were living with a partner and agriculture was their main activity. Household size ranged from 2 to 15 and parity from 1 to 17. More than half of the mothers had 4 or more children. For 586 households (87.5%) who produced rice, the median duration covered by rice production was 4 months. Regarding nutritional status, less than 10% of mothers had in poor nutritional status, based on either their size (<145cm), their MUAC (<220mm) and/or their presence of anemia (Hb <120g/l). According to the BMI, 13% of mothers had mild undernutrition (17 ≤BMI <18.5), 3% had moderate undernutrition (16 ≤BMI <17) and 1% had severe undernutrition (BMI < 16). In contrast, 5.8% of mothers were overweight (25 ≤BMI<30) and 1.2% were obese (BMI≥30).

**Table 1:** Mothers' social characteristics and nutritional status

Social profile (n=670)	%	Social profile (n=670)	%
**Age (years)**	33 (±7)^a^	Breastfeeding	
**Education level**		Yes	42.2
Illiterate & primary	72.7	No	57.8
Secondary 1^st^ cycle	22.4	Household size	
Secondary 2^nd^ cycle	4.9	< 6	46.3
**Marital status**		≥ 6	53.7
Couple	77.6	Rice availability (/12 months)	
Single	22.4	≤ 6 months	67.3
**Occupation**		> 6 months	20.2
Farmer	90.3	No producer	12.5
Other	9.7	Time to go to the market (h)	
**Parity**		<½	10.4
1-3	43.7	½ - 1	51.5
4 and +	56.3	>1	38.1
**Birth interval (months)**		Market attendance (/week)	
< 24	14.2	< 1	18.6
≥ 24	85.8	= 1	54.8
**Height (cm)**		> 1	26.6
< 145	8.8	MUAC (mm)	
≥ 145	91.2	< 220	8.7
**BMI (kg/m^2^)**		≥ 220	91.3
<18.5	17.0	Hemoglobin (g/l)	
18.5-24.9	76.0	<120	7.2
≥ 25	7.0	≥ 120	92.8

BMI: Body Mass Index, MUAC: Mid Upper Arm Circumference

a Mean (±SD)

**Consumed food**: According to the 24-hour recall, 93% of mothers ate 3 meals on the day before the survey. They all had dinner (100%) but 2.5% and 4.8% missed breakfast and lunch respectively. Three out of 10 mothers had eaten a snack at least once during the day: 8.5% in the morning, 13.7% in the afternoon and 8.2% both in the morning and the afternoon. Starches were among frequently consumed food, i.e. rice (100%), sweet potato (50%), cassava (30%) and potatoes (18%). Next came green-leafy vegetables: “anandrebaka” (58%), “petsay” (16%) and sweet potato leaves (15%). Legumes and meat were less frequently consumed: beans (13%), fish (12%), zebu meat (7%) and pork (5%). Fruits and dairy were rarely consumed (<5%).

### Dietary profile

**Figure 1:**
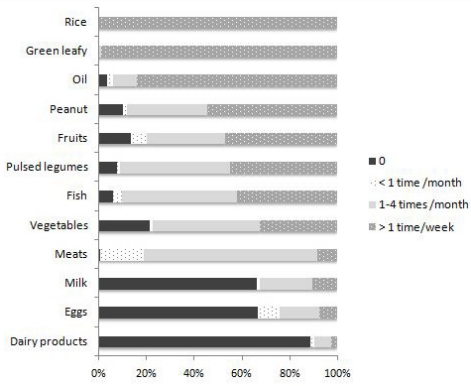
Distribution of mothers according to the frequency of food intake over the last 3 months

**Frequency of food consumption**: Figure 1 shows results of the Food Frequency Questionnaire for the last 3 months before the survey, the post-harvest season. We observed that 99% of mothers ate rice every day: once a day for 17% and thrice a day for 30%. Almost all mothers (99%) ate green-leafy vegetables more than once per week, and 59% did so every day. The following foods were eaten more than once a week, by nearly half of mothers: peanuts (54%), fruits (47%), legumes (45%) and fish (42%). Meat consumption was low and the proportion of mothers who have never consumed dairy products, milk and eggs was high. Iodized salt was used every day by only 9.7% of mothers. It was used irregularly by 19.4%, never used by 31.0% and 39.9% of mothers did not know if the salt used was iodized or not.

**Figure 2:**
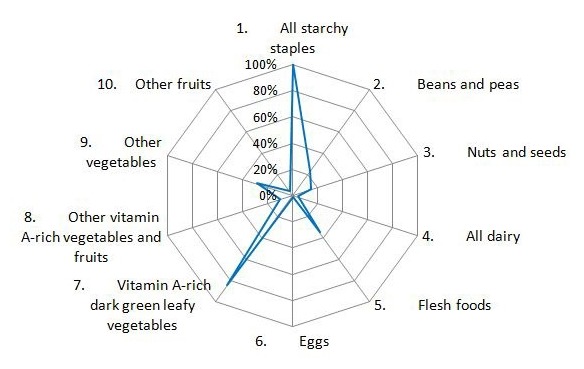
Proportion of mothers who consumed food from the 10 food groups according to the 24-hour recall

**Dietary diversification**: Figure 2 shows intake frequencies for the 10 food groups recommended for estimating the women's dietary diversity (according to the 24 hours recall). Starches and dark green-leafy vegetables rich in vitamin A were the most frequently food groups eaten by mothers. The dietary diversity score of mothers ranged from 1 to 7 and 88% of mothers have a dietary diversity score below 5.

**Dietary diversity and characteristics of mothers**: Table 2 shows the results of the bivariate analysis that looked at the link between low dietary diversity score and characteristics of mothers. Low dietary diversity score (< 5) was significantly more common among farming mothers, with low education level, whose parity was at 4 or above with a birth interval higher than 24 months and a household size equal to or greater than 6. Regarding economic factors, the proportion of mothers with low dietary diversity was significantly higher when annual rice production covered less than half the year. This proportion decreased in parallel with the time taken to go to the market, the household's market attendance and the score of movable property possession. Table 3 shows the results of the multivariate analysis. After adjusting for the other variables in the model, the conditions associated with low dietary diversification were: low education, high parity (4 or more), birth interval above 24 months. Household characteristics associated with low dietary diversity were: insufficient rice production (covering less than half of the year), low market attendance and decreased movable property possession score.

**Table 2:** link between low Dietary Diversity Score (DDS<5) and socio-economic characteristic and nutrition status of mothers

	Nb (n=670)	% mothers with DDS<5	OR [CI 95%]	p
**Age (years)**				
18-29	206	85.0	1	0.306
30-39	322	89.4	1.50 [0.89-2.53]	
40-45	142	88.3	1.30 [0.69-2.46]	
**Education level**				
Illiterate & primary	487	92.2	7.68 [3.56-16.61]	<0.001^a^
Secondary 1^st^ cycle	150	79.3	2.50 [1.18-5.57]	
Secondary 2^nd^ cycle	33	60.6	1	
**Marital status**				
Couple	520	87.1	1	0.342
Single	150	90.0	1.33 [0.74-2.41]	
**Occupation**				
Farmer	605	88.6	1.94 [1.01-3.75]	0.045
Other	65	80.0	1	
**Parity**				
1-3	293	82.2	1	<0.001
4 and +	377	92.0	2.50 [1.54-4.03]	
**Breastfeeding**				
Yes	283	87,6	1	0.931
No	387	87,9	1.02 [0.64-1.63]	
**Birth interval (months)**				
< 24	95	78.9	1	0.006
≥ 24	575	89.2	2.21 [1.26-3.86]	
**Household size**				
< 6	310	84.2	1	0.010
≥ 6	360	90.3	1.86 [1.16-2.98]	
**Rice availability (/year)**				
≤ 6 months	451	91.4	3.15 [1.88-5.29]	<0.001
> 6 months	135	77.0	1	
Non producer	84	85.7	1.79 [0.86-3.71]	
**Market attendance (/week)**				
< 1	125	95.2	5.56 [2.28-13.60]	<0.001^a^
= 1	367	89.9	2.50 [1.53-4.09]	
> 1	178	78.1	1	
**Time to go to the market (h)**				
<½	70	72.8	1	<0.001^a^
½ - 1	345	88.4	2.84 [1.53-5.29]	
>1	255	87.8	3.76 [1.91-7.41]	
**Movable property possession score**				
Low	218	95.9	7.20 [3.45-15.01]	<0.001^a^
Medium	228	91.2	3.22 [1.85-5.60]	
High	224	76.3	1	
**Agricultural score**				
Low	351	92.6	2.66 [1.37-5.13]	<0.001
Medium	222	82.4	0.99 [0.53-1.87]	
High	97	82.5	1	
**Farming animals score**				
Low	254	87.0	1.18 [0.70-1.98]	0,107
Medium	195	91.8	1.96 [1.04-3.69]	
High	221	85.1	1	
**MUAC (mm)**				
<220	58	89.7	1.23 [0.51-2.96]	0.645
≥220	612	87.6	1	
**BMI (kg/m^2^)**				
<18,5	114	92.1	1.76 [0.85-3.64]	0.120
≥18,5	556	86.9	1	
**Anemia**				
Yes	48	89.6	1.22 [0.47-3.16]	0.689
No	622	87.6	1	

DDS: Dietary diversity Score, OR: Odds Ratio, CI: Confidence interval, MUAC: Mid Upper Arm Circumference, BMI: Body Mass Index

a : chi square for trend

**Table 3:** Adjusted OR of low dietary diversification (Dietary Diversity Score <5) (588 mothers with low dietary diversity score)

Variable	Nb (n=670)	DDS <5 AOR [CI 95%]	p
**Education level**			
Illiterate & primary	487	3.80 [1.58-9.02]	0.003
Secondary 1^st^ cycle	150	1.87 [0.78-4.48]	
Secondary 2^nd^ cycle	33	1	
**Parity**			
1-3	293	1	0.007
4 and +	377	2.09 [1.22-3.56]	
**Birth interval (months)**			
< 24	95	1	<0.001
≥ 24	575	4.01 [2.08-7.74]	
**Rice availability (/year)**			
≤ 6 months	451	2.33 [1.30-4.17]	0.013
> 6 months	135	1	
no produce	84	1.25 [0.54-2.91]	
**Market attendance (/week)**			
< 1	125	4.20 [1.63-10.83]	<0.001
=1	367	2.43 [1.41-4.18]	
> 1	178	1	
**Movable property possession score**			
Low	218	4.87 [2.15-11.04]	<0.001
Medium	228	2.26 [1.21-4.21]	
High	224	1	

AOR: Adjusted Odds Ratio, CI: Confidence interval

H-L p=0.097

Not included in the model because not significant: occupation, household size, agricultural score, farming animals score.

## Discussion

The results of the study provided a general overview of the feeding of mothers in the study area. They have a diet based on rice and green leaves, poor in foods rich in protein and poorly diversified. Rice is the staple food of the majority of Malagasy and is considered a “king product”. The frequency of daily consumption of rice depends on its availability and a low frequency of consumption generally implies a food safety problem or an unfavorable economic situation. The ideal for the population is to take a meal three times a day. The frequency of rice consumption still seems to be important during this post-harvest period because 30% of mothers ate it 3 times a day and 53% twice a day. The population of the region is composed of 90% of agricultural households and rice cultivation remains the most practiced [7,14]. Over one year, the availability of rice (production) didn't exceed 6 months for 79.8% of the studied households. When rice is not available or is insufficient, the rural population buys or replaces it with tubers (sweet potato and cassava) [15]. In the study area, cassava and sweet potatoes were placed second and third after rice cultivation [14]. People usually take rice with green leaves or legumes, which increases the chance of a more diverse diet. On the other hand, sweet potato and cassava are consumed without accompaniment. Despite the high frequency of consumption of energy foods, data available at national level showed that in 2013, 58% of Malagasy people have a very poor energy consumption (bringing less than 2250 kcal per day). For 60% of cases, more than 85% of calories come from carbohydrates (rice, sweet potato, cassava, etc.), instead of 55% to 75% according to the WHO recommendation for nutritional balance [7,16]. Results from a series of studies on women's dietary diversity in 5 low-resource countries showed a high proportion of calories from carbohydrates: 86% in Mozambique and 82% in Bangladesh [17].

The results showed a lower frequency of consumption of foods rich in protein: 55%, 58% and 91% of mothers respectively ate legumes, fish and meat less than once a week. In the Amoron'i Mania Region, the post-harvest period does not coincide with that of legume production. On the other hand, the availability of freshwater fish increases because of the possibility of collecting them in the rice fields after the rice harvest. Lower market attendance and lack of money explain the low consumption of meat. The small family farm is not intended for self-consumption. Meat consumption in the study population is relatively low compared to other low-resource countries. In the Philippines, Mali and Burkina Faso, the proportion of women of reproductive age who consumed meat, according to the 24-hour recall, exceeds 90% [17]. In Mozambique, this proportion was estimated at 45%, which is always higher than the 35% found among mothers in the study area [17]. As for the consumption of vegetables, it remains low except for the green leaves that exist throughout the year but whose type varies according to the season. In general, people collect those that are available in the fields and during the period studied, the “anandrebaka” sheet consumed by 58% of the mothers is the most found in the fields. Dark green leaves have the characteristic of being rich in vitamin A and iron but it is known that the bioavailability of iron is not better for foods of vegetable origin. Consumption of fruit, eggs and milk and dairy products is rare. The availability of fruit depends on the season [18]. At the time of the survey, no fruit was available in the fields. Nearly 9 out of 10 women have a poorly diversified diet (dietary diversity score <5). The dietary diversity score in two categories is an indicator of the quality of the diet and essentially reflects the adequacy in micronutrients [12]. This is why we have not found any relationship with the nutritional status of mothers. In terms of food group, a similarity between the results of the frequency of consumption and the 24-hour recall is observed, even if the latter does not claim to estimate the usual consumption. The exploratory study conducted prior to this study found that food consumption patterns of mothers did not experience a significant weekly variation. The results of a series of studies on dietary diversity of women in 5 low-resource countries yielded an average dietary diversity score ranging from 3.8 in Mozambique to 5.5 in Mali (indicator calculated with 9 food groups according to the previous recommendation) [17]. According to the results of the multivariate analysis, the characteristics of mothers with low dietary diversification scores are low education, high parity, long birth interval (≥24 months), less rice availability (≤6 months), lower market attendance and low property ownership score. All of these characteristics, except for the long birth interval, reflect an unfavorable social or economic situation that results in reduced access to diversified foods. According to the results of the studies, these adverse socio-economic conditions are associated with low dietary diversity in low-income countries [19].

Social inequalities observed in mothers are factors that determine their dietary practice. Mothers with a low level of education are very numerous in the present study, with 73% who did not go beyond primary level. They are more frequent in the group with a low dietary diversity score and this is confirmed by the results of other studies [19,20]. The mother's level of education could influence their knowledge of dietary diversification as well as the willingness and effort to diversify the diet. High parity goes hand in hand with a high household size. It implies an increase in household food requirements and a risk of having a diet that is poor in quality and quantity. Most households in the study area live on subsistence farming, have limited arable land and insufficient production [14]. Households with a high size are more vulnerable to food insecurity [21]. As for market attendance, the explanation of its role on the variation of food consumed requires additional information such as distance from the market and the reason for going there (selling, buying, meeting friends or relatives, simple habit, etc). However, frequenting the market increases the chance of obtaining a variety of foods. Market access is a component of household food security and better market access is associated with good dietary diversification [22,23]. Regarding the birth interval, all mothers with short intervals still have children under 5 years of age. Having young children in the household may be conducive to good dietary diversification. Special attention to feeding children and nutritional counseling received by mothers through Infant and Young Child Feeding (IYCF) projects could have beneficial effects on the diversification of diet of the whole family [24,25]. The identified factors could be related to household food insecurity. In a low resource country such as Madagascar, the possibility of diversifying food is dictated mainly by household food security [26,27]. For farm households, everything depends on their production and crop variability and cultivated area plays a major role [22]. In this study, most mothers do not know the area cultivated by their household. However, the data available in 2013 showed that the median economic farming area exploited per household was fairly small, i.e. 0.4 hectares for the Amoron'i Mania Region. It is the lowest value of the 22 regions of the country (1 hectare for the whole country) [14]. In the fight against maternal and infant malnutrition, improving the availability and accessibility of household food through nutrition-sensitive agriculture programs has been effective [28]. It is a strategy tailored to the context of the study area where almost all people live on subsistence farming and the food consumed will depend to a large extent on available crops.

## Conclusion

The present study showed that the food consumption of mothers in the study area did not allow them to ensure a good diet. Their diet was poorly diversified and low in protein. Unfavorable socio-economic conditions are associated with this low food diversification and expose the population to a food insecurity problem.

### What is known about this topic

- Inadequate diet is one of the main causes of malnutrition;
- In developing countries, food practice depends mainly on food security;
- Standard on Women Dietary Diversity Score (WDDS) according to the results of several studies conducted in developing countries (new recommendation in 2016).

### What this study adds

- Availability of baseline data on women's dietary diversity in Madagascar (required for monitoring and evaluation of programmes to fight maternal malnutrition);
- Knowledge of the specificity of mothers' feeding practices in the study area;
- Identify the determinants of food practice, which helps to guide the choice of strategies for improving nutrition.
